# Defects in mTORC1 Network and mTORC1-STAT3 Pathway Crosstalk Contributes to Non-inflammatory Hepatocellular Carcinoma

**DOI:** 10.3389/fcell.2020.00225

**Published:** 2020-04-07

**Authors:** Ting Li, Guohong Zhang, Linlin Wang, Susu Li, Xiaoping Xu, Yi Gao

**Affiliations:** ^1^Department of Hepatobiliary Surgery II, Zhujiang Hospital, Southern Medical University, Guangzhou, China; ^2^Department of Pathology, Shantou University Medical College, Shantou, China; ^3^Institute of Regenerative Medicine, Zhujiang Hospital, Southern Medical University, Guangzhou, China; ^4^Department of Oncology, Nanfang Hospital, Southern Medical University, Guangzhou, China; ^5^Department of Cell Biology, School of Basic Medical Sciences, Southern Medical University, Guangzhou, China; ^6^Artificial Organs and Tissue Engineering Centre of Guangdong Province, Guangzhou, China; ^7^State Key Laboratory of Organ Failure Research, Southern Medical University, Guangzhou, China

**Keywords:** carcinogenesis, mouse model, non-inflammation, gene network, metabolic disorder

## Abstract

**Background and Aims:**

Mammalian target of rapamycin complex 1 (mTORC1) is frequently hyperactivated in hepatocellular carcinoma (HCC). Cases of HCC without inflammation and cirrhosis are not rarely seen in clinics. However, the molecular basis of non-inflammatory HCC remains unclear.

**Methods:**

Spontaneous non-inflammatory HCC in mice was triggered by constitutive elevation of mTORC1 by liver-specific *TSC1* knockout (*LTsc1KO*). A multi-omics approach was utilized on tumor tissues to better understand the molecular basis for the development of HCC in the *LTsc1KO* model.

**Results:**

We showed that *LTsc1KO* in mice triggered spontaneous non-inflammatory HCC, with molecular characteristics similar to those of diethylnitrosamine-mediated non-cirrhotic HCC. Mitochondrial and autophagy defects, as well as hepatic metabolic disorder were manifested in HCC development by *LTsc1KO*. mTORC1 activation on its own regulated an oncogenic network (DNA-damage-inducible transcript 4, nuclear protein 1, and fibroblast growth factor 21), and mTORC1–signal transducer and activator of transcription pathway crosstalk that altered specific metabolic pathways contributed to the development of non-inflammatory HCC.

**Conclusion:**

Our findings reveal the mechanisms of mTORC1-driven non-inflammatory HCC and provide insight into further development of a protective strategy against non-inflammatory HCC.

## Introduction

Hepatocellular carcinoma (HCC) is the fifth most common cancer in men and the seventh in women, and is now the second most common cause of cancer-related death worldwide (Bosetti et al., 2014; Inarrairaegui et al., 2018). Patients with advanced HCC have limited treatment options, and chemotherapy provides minimal survival benefit. Lack of knowledge of the mechanisms of HCC is one of the important factors that limit the development of HCC treatment. Therefore, understanding the underlying mechanisms of HCC is still urgently needed to explore novel therapeutic options.

Hepatitis B or hepatitis C is the leading risk factor for HCC, and other risk factors include obesity, diabetes and related nonalcoholic fatty liver disease (NAFLD) (Yu and Yuan, 2004; Mittal and El-Serag, 2013). Chronic inflammation is closely associated with persistent hepatic injury and concurrent regeneration, leading to sequential development of fibrosis, cirrhosis, and eventually HCC (Bishayee, 2014). However, cases of HCC in the absence of inflammation and cirrhosis are not rare in clinics (Guzman et al., 2008; Mohamad et al., 2016). Most studies have focused on the mechanisms of chronic-inflammation-based HCC, while few studies have been conducted on the molecular basis of non-inflammatory HCC in animal models (Moriya et al., 2001). The mechanisms of HCC in the absence of inflammation are still undefined and need further investigation.

The mammalian target of rapamycin (mTOR) pathway is aberrantly up-regulated in up to 50% of HCCs (Bhat et al., 2013). The mTOR kinase nucleates two distinct protein complexes termed mTOR complex 1 (mTORC1) and complex 2 (mTORC2). The mTOR pathway regulates key cellular functions linked to the promotion of cell growth and metabolism (Guertin and Sabatini, 2007). Various signaling pathways upstream of mTORC1 stimulate its activity through inhibition of the tuberous sclerosis (TSC) 1–TSC2 complex. Disruption of this complex, through the loss of TSC1 or TSC2, results in ectopic activation of mTORC1 (Laplante and Sabatini, 2009). Dysfunction of hepatic TSC1, a suppressor of mTOR signaling, occurs in patients with hepatitis B, hepatitis C or insulin resistance, which is a risk factor for HCC development (Lee et al., 2008; Totoki et al., 2011; Yen et al., 2012; Ho et al., 2017). Menon et al. (2012) detected sporadic HCC development in a liver-specific *TSC1* knockout (*LTsc1KO*) mouse model and proposed that the liver damaging, inflammatory cycles of necrosis and regeneration may contribute to the development of HCC in *LTsc1KO* mice. However, in this study, we did not find any obvious histological evidence of inflammation, necrosis and fibrosis in *LTsc1KO* HCC mice. These mice are a good model to investigate the underlying molecular mechanisms of HCC development in the absence of inflammation. We utilized a multiomics strategy including transcriptomics, cytokine proteomics, genomics and metabolomics, to determine the comprehensive molecular basis for without long-term hepatic inflammation, necrosis, or fibrosis.

Our findings demonstrate that inflammation and fibrosis are not prerequisites in HCC development triggered by *TSC1* deficiency. Non-inflammatory HCC developed by *LTsc1KO* displayed molecular characteristics that were similar to those of diethylnitrosamine (DEN)-mediated non-cirrhotic HCC (Chen et al., 2015). Mitochondrial and autophagy defects, as well as hepatic steatosis were manifested in HCC. mTORC1 activation on its own regulated a gene network, including DNA-damage-inducible transcript 4 (Ddit4), nuclear protein 1 (Nurp1) and fibroblast growth factor 21 (FGF21), and mTORC1–signal transducer and activator of transcription pathway crosstalk that altered specific metabolic pathways contributed to the development of non-inflammatory HCC.

## Materials and Methods

### Generation of Mice

Mice carrying the *Tsc1*^fl^ allele in the FVB background have been previous described (Yecies et al., 2011). *LTsc1KO* mice were generated by crossing *TSC1*^fl/*fl*^ mice with *Alb-Cre* mice (C57BL/6J background) obtained from Jackson Laboratory (Farmington, CT, United States) (Kwiatkowski et al., 2002). The specificity of recombination was confirmed by PCR using primers flanking the floxed allele. The primer sequences are listed in Supplementary Table S1. All procedures involving mice was approved by the Southern Medical University Animal Care and Use Committee. Mice importing, transporting, housing, and breeding were conducted according to the recommendations of “The use of non-human primates in research.”

### Mouse Diets and Treatments

The mice were housed in plastic cages at a controlled temperature of 22 ± 1°C on a 12 h light/12 h dark cycle with lights on from 06:00 to 18:00 h. Standard rodent chow and water were provided *ad libitum* throughout the entire feeding period. All of the animal experiments were approved by the Animal Ethics Committee of Southern Medical University (approval number SYXK 2011-0074) and performed in accordance with animal ethics guidelines and approved protocols. Mice aged 5 months were subjected to normal saline (NS) or rapamycin gavage. For rapamycin treatment, mice were administered by oral gavage 5 mg/kg/day rapamycin until killing at 10 months of age, and the same volume of NS was administered to control mice. All mice were killed prior to their daily feeding.

### Statistical Analysis

Continuous variables were compared using independent sample *t*-tests or one-way analysis of variance, with the data expressed as mean ± SEM. Significant differences are indicated in the figure legends, unless otherwise indicated.

Other detailed experimental procedures are described in Supplementary Material.

## Results

### *LTsc1KO* Mice Developed Spontaneous HCC Without Systemic Glucose Tolerance

**FIGURE 1:**
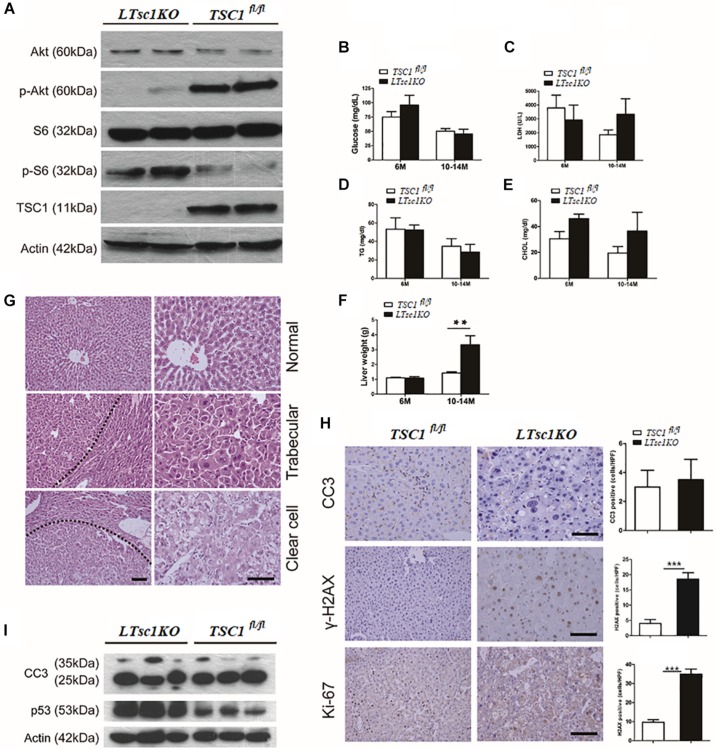
LTsc1KO mice developed spontaneous HCC. **(A)** Immunoblot analysis of isolated hepatocytes from *LTsc1KO* and *TSC1*^fl/fl^ mice with TSC1, S6, Akt, and their phosphorylation antibodies to confirm activation of mTORC1. **(B)** Blood glucose levels in 6- and 10–14-month-old *LTsc1KO* and *TSC1*^fl/fl^ mice, respectively (*n* = 45, ±SEM). **(C–E)** Serum LDH, cholesterol, and triglyceride levels were observed in 6- and 10–14-month-old *LTsc1KO* and *TSC1*^fl/fl^ mice, respectively. **(F)** Liver weights in 6- and 10–14-month-old *LTsc1KO* and *TSC1*^fl/fl^ mice (*n* = 45, ±SEM), and liver weight was increased in 10–14-month-old *LTsc1KO* comparing with *TSC1*^fl/fl^ mice (**P < 0.01). **(G)** Liver sections showing histological types of tumor in 10–14-month-old *LTsc1KO* mice. The tumor areas are marked in low magnification and high magnification to show the histological features clearly. **(H)** Cleaved caspase-3 (CC3), p-H2AX, and Ki67 staining of liver sections in 10–14-month-old *LTsc1KO* and *TSC1*^fl/fl^ mice. Immunohistochemically positive cells were quantified, and results are shown in the bar graphs (*n* = 19, ± SEM, ***P < 0.001). **(I)** Hepatic CC3 and p53 protein expression in *LTsc1KO* and *TSC1*^fl/fl^ mice.

We generated *LTsc1KO* mice by crossing *TSC1*^fl/fl^ with *Alb-Cre* mice. Deletion of the hepatic *TSC1* allele was confirmed by polymerase chain reaction (PCR) of genomic DNA (Supplementary Figure S1A). Immunoblot analysis of *LTsc1KO* liver revealed constitutively active mTORC1 signaling in the liver, as indicated by TSC1 and the best-characterized substrates of mTORC1 S6 phosphorylation (pS6). *LTsc1KO* mice displayed a strong reduction in phospho-Ser473-PKB/Akt (p-Akt). As described previously (Yecies et al., 2011), in the present study, the *LTsc1KO* livers display attenuation of Akt signaling due to a mTORC1-dependent negative feedback loop (Figure 1A). Surprisingly, serum parameters showed that *LTsc1KO* mice displayed unchanged blood glucose, triglyceride, lactate dehydrogenase (LDH), and cholesterol levels relative to *TSC1*^fl/fl^ mice, indicating a normal insulin response in the *LTsc1KO* mice (Figures 1B–E). Compared with the *TSC1*^fl/fl^ mice, *LTsc1KO* mice did not have any decrease in body weight by 6 and 10–14 months.

There were no detectable tumors in the *LTsc1KO* mice by 6 months, while 31 of 45 (68.89%) *LTsc1KO* mice spontaneously developed HCC by 10–14 months. An average of three macroscopic tumors without encapsulation per liver was detected. Multifocal tumors were randomly distributed in all liver lobes (Supplementary Figure S1B). Consistently, these *LTsc1KO* mice exhibited increased liver weight by 10–14 months (Figure 1F).

Previously, tumor developed by *TSC1* deficiency has been considered as benign with a high rate of apoptosis (Wataya-Kaneda et al., 2001). In the present study, histopathological features of HCC in *LTsc1KO* mice revealed large cells with enlarged and hyperchromatic nuclei and ballooning with weakly eosinophilic staining, which contained numerous microvesicular vacuoles in cytoplasm (Figure 1G). *LTsc1KO* did not induce significant apoptosis, as measured by cleaved caspase 3. Phosphorylated histone-2AX (γH2AX)-positive hepatocytes, was increased in *LTsc1KO* livers (Figure 1H). p53 level was increased in livers of *LTsc1KO* mice. These data demonstrate that loss of *TSC1* results in dramatic accumulation of p53 in response to the p53-mediated DNA damage-response pathway (Figure 1I).

### Hepatic Necroinflammation Was Not a Prerequisite for Spontaneous HCC in *LTsc1KO* Mice

**FIGURE 2:**
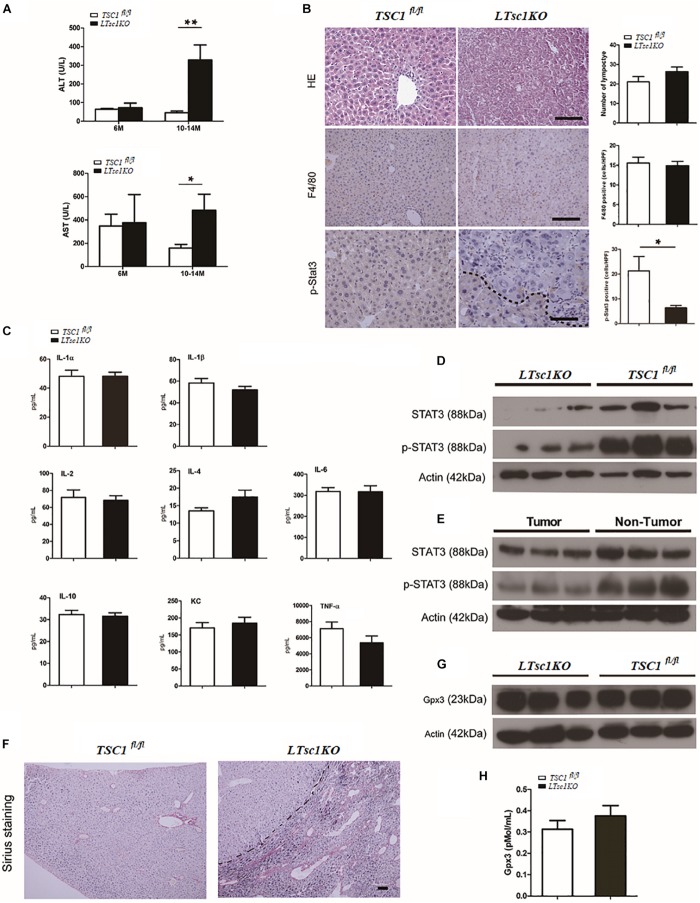
Spontaneous HCC development in *LTsc1KO* mice lacked necroinflammation and fibrosis. **(A)** Serum ALT and AST levels in 6- and 10–14-month-old *LTsc1KO* and *TSC1*^fl/fl^ mice (*n* = 29, ±SEM). ALT and AST only increased in 10–14-month-old *LTsc1KO* compared with *TSC1*^fl/fl^ mice (**P = 0.006, and *P = 0.046). **(B)** H&E, F4/80 and p-STAT3 immunohistochemical staining of liver sections in 10–14-month-old *LTsc1KO* and *TSC1*^fl/fl^ mice. Immunohistochemically positive cells were quantified, and results are shown in the bar graphs (*n* = 19, ± SEM, *P < 0.05). **(C)** Hepatic levels of inflammatory cytokines in 10–14-month-old *LTsc1KO* and *TSC1*^fl/fl^ mice (*n* = 19, ±SEM). **(D)** Immunoblot analysis of hepatic STAT3 and p-STAT3 in *LTsc1KO* and *TSC1*^fl/fl^ mice aged 10–14 months. **(E)** Immunoblot analysis of hepatic STAT3 and p-STAT3 in tumor and non-tumor areas of *LTsc1KO* mice. **(F)** Fibrosis was analyzed by staining liver sections with Sirius red in 10–14-month-old *LTsc1KO* and *TSC1*^fl/fl^ mice. **(G,H)** Serum and hepatic GPX3 level in *LTsc1KO* and *TSC1*^fl/fl^ 10–14-month-old mice (*n* = 14, ±SEM).

We found marked elevation of serum ALT and AST in *LTsc1KO* mice at 10–14 months but not at 6 months, indicating no long-term hepatic damage in *LTsc1KO* mice (Figure 2A). No significant changes in the number of infiltrative lymphocytes were observed in *LTsc1KO* mice as compared with *TSC1*^fl/fl^ mice. F4/80 staining suggested that the number of macrophages (Kupffer cells) remained unchanged in the liver from *LTsc1KO* mice at 6 and 10–14 months (Figure 2B).

To explore further whether there was chronic hepatic inflammation in *LTsc1KO* mice, we conducted a Bio-Plex mouse cytokine 23-plex assay to detect simultaneously 23 cytokines, chemokines and growth factors. It has been proved that increased IL-6 production results in activation of the oncogenic transcription factor STAT3 in liver, and TNF-α is a major adipose- derived cytokine and potent activator of pro-oncogenic pathways, including mTOR. The cytokine assay revealed no increased expression of inflammatory cytokines, including TNF-α and IL-6 (Figures 2C and Supplementary Figure S1C). Taken together, these results indicated that, the development of HCC in *LTsc1KO* mice was not dependent on long-term injury and necroinflammation.

We found that levels of STAT3 phosphorylation decreased in the livers of *LTsc1KO* mice as compared with *TSC1*^fl/fl^ mice, and in tumors as compared with non-tumor areas of the liver in *LTsc1KO* mice (Figures 2D,E). It is worth noting that activity of serum and cellular GPX3 in *LTsc1KO* mice was similar to that in *TSC1*^fl/fl^ mice (Figures 2G,H). We therefore propose that HCC without necroinflammation developed possibly through inactivation of STAT3 and/or other mutations. However, we cannot completely exclude the possibility of additional mechanisms by which mTOR affects STAT3 phosphorylation.

### Spontaneous HCC in *LTsc1KO* Mice Displayed Non-cirrhotic and Chemical Carcinogenic Characteristics

**FIGURE 3:**
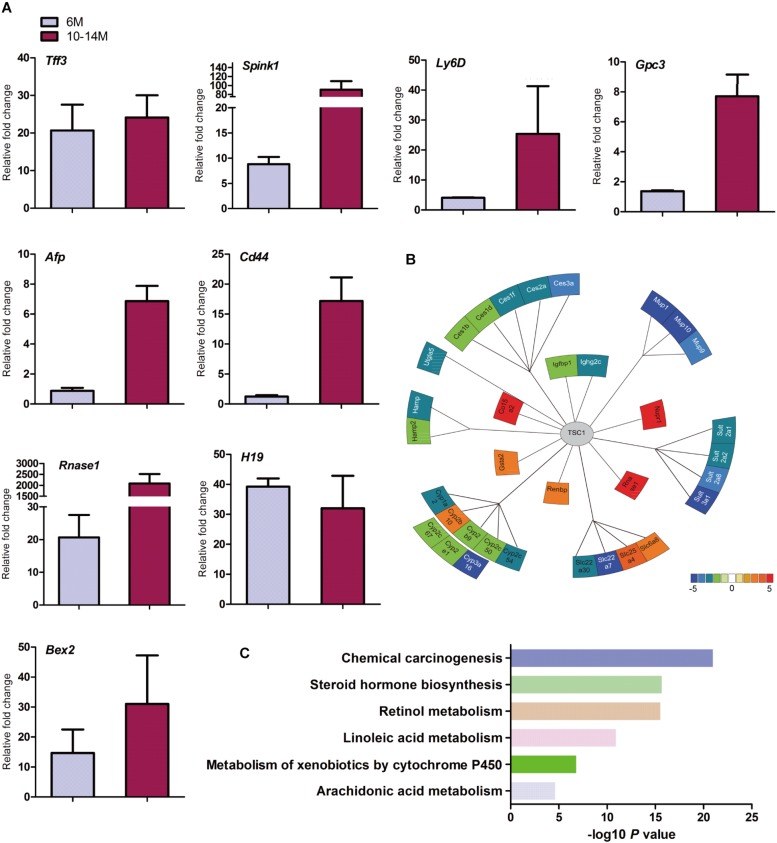
Spontaneous HCC in *LTsc1KO* mice showed expression of genes commonly induced in chemical HCC. **(A)** qRT-PCR showing increased hepatic mRNA levels for expression of genes that are frequently induced in *LTsc1KO* mice aged 6 and 10–14 months, which were similar to human DEN-mediated HCC, such as *Tff3*, *Gpc3*, and *Ly6d* (*n* = 19, ±SEM). **(B)** Illustration of differentially expressed genes in HCC. Heat-map indicates mean values of differential expression for each gene. **(C)** Histographs of differentially expressed genes by pathway analysis.

Cirrhosis is not always a prerequisite of HCC development and this might particularly apply to the HCC associated with metabolic abnormality. Sirius red staining showed that except for increased blood vessels, no obvious fibrosis was discovered, which demonstrated that hepatic mTORC1 activation did not induce liver fibrosis (Figure 2F). To investigate the molecular characteristics of HCC in *LTsc1KO* mice, we conducted RNA sequencing (RNA-seq) on mice at 6 and 10–14 months. The pattern of gene expression in the livers of *LTsc1KO* mice was also induced in non-cirrhotic DEN-mediated HCC, including *Tff3, Ly6d, Gpc3*, and *Afp* (Figure 3A), and several other fetal genes that were induced in human HCC, such as *Bex2, Spink1*, and *Rnase1*. The change in Tff3 protein was confirmed by western blotting (Figure 5C).

Further analysis of the RNA-seq data revealed that the aberrant expression of enzyme genes were mainly responsible for detoxification (Figure 3B), including cytochrome P450 (CYP450), carboxylesterases (Ces), sulfotransferases (Sults), and UDP-glucuronosyltransferases (Ugts). The activities of xenobiotic metabolizing enzymes are required for activation (toxication) of important carcinogens. CYP450 enzymes are key players in the phase-I-dependent metabolism of xenobiotics. We found reduced expression of Cyp1a2, Cyp2b9, Cyp2c50, Cyp2c54, Cyp2c67, Cyp2e1, and Cyp3a16, and increased expression of Cyp2b10 in the livers of *LTsc1KO* mice. Further gene ontology (GO) enrichment analysis revealed that differentially expressed genes were significantly enriched in biological process of chemical carcinogenesis (Figure 3C). We suggest that the first stage of carcinogenesis induced by mTORC1 activation is similar to that induced by chemical carcinogens, which results in cells with altered metabolic responsiveness and a proliferative advantage over the surrounding normal cells.

### *LTsc1KO*-Induced Spontaneous HCC Was Also Characterized by Metabolic Disorder

**FIGURE 4:**
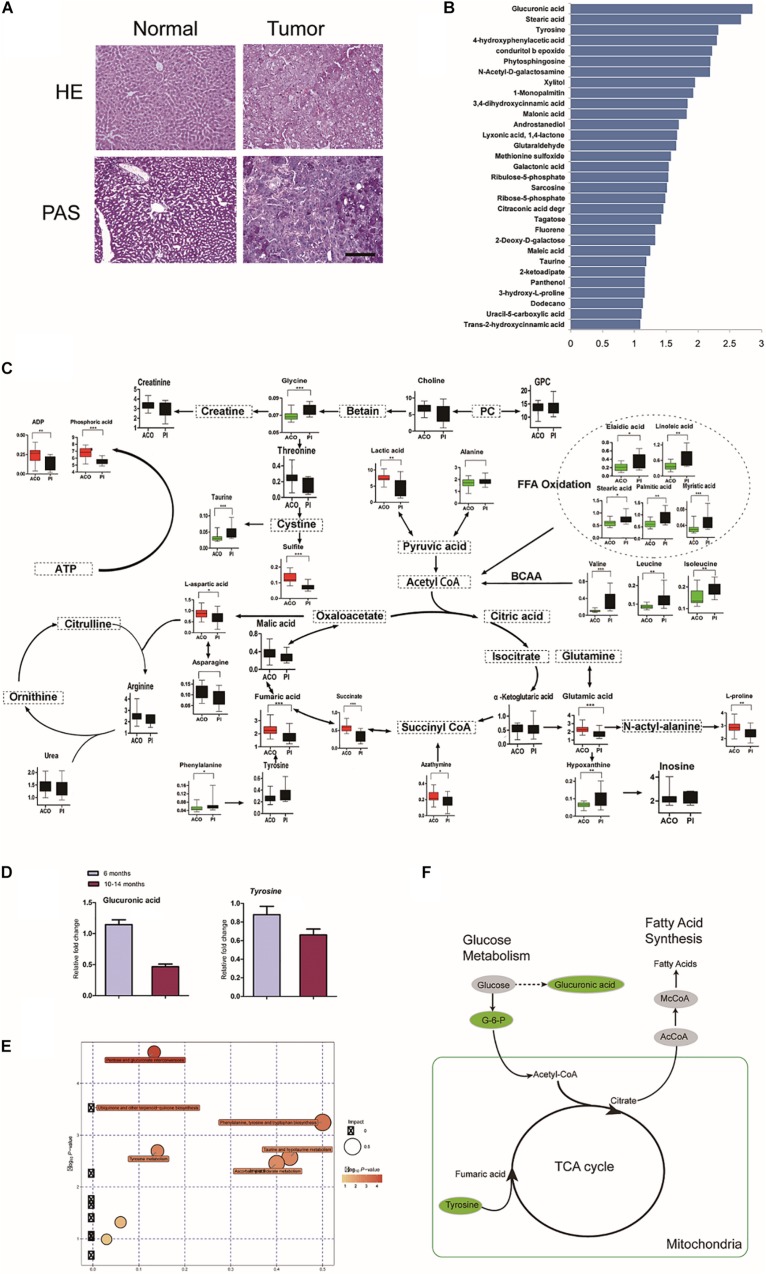
Characterization of *LTsc1KO* liver-bearing carcinoma by glucuronic acid loss and metabolic disorder. **(A)** Liver sections were stained for glycogen with PAS. **(B)** Representative pictures of the significantly changed metabolites by –log10 *P*-value. **(C)** Metabolic pathway map in *LTsc1KO* mice aged 10–14 months. Green, decrease; red, increase. **(D)** Concentrations of hepatic glucuronic acid and tyrosine in *LTsc1KO* and *TSC1*^fl/fl^ mice aged 6 and 10–14 months (*n* = 19, ±SEM). **(E)** A differential score was calculated for the KEGG pathway. Size of dots indicates the impact of the pathway and the color indicates pathways with a score of – log10 *P*-value. **(F)** Metabolic shifts in the tricarboxylic acid cycle in *LTsc1KO* mice aged 10–14 month. Green, decrease; gray, not measured.

We checked whether metabolic disorder occurred. Periodic acid-Schiff (PAS) staining confirmed that there was less glycogen in HCC foci than adjacent hepatic tissues (Figure 4A). The metabolome was investigated in livers at 6 and 10–14 months (Figures 4B,C). Consistent with reduction of Ugts1a by RNA-seq, the metabolomic changes were loss of glucuronic acid, with dramatically altered cellular metabolism at 10–14 months compared with 6 months (Figure 4D).

Pathway analysis revealed that many related metabolic pathways that influence various biological processes, such as pentose and glucuronate interconversions (carbohydrate metabolism subcategory), and the biosynthesis of ubiquinones (Figure 4E). Integrated omics profiling revealed that carcinogenesis of spontaneous HCC in *LTsc1KO* mice was similar to chemical carcinogenesis. Therefore, HCC induced by *LTsc1KO* was characterized by decreased glucose and TCA cycle disorder (Figure 4F). These findings suggest that specific metabolic changes, commonly seen in human hepatic carcinoma, might contribute to tumor progression in this model.

### Hepatocarcinogenesis Was Subsequent to Defects Upstream and Downstream of mTORC1

**FIGURE 5:**
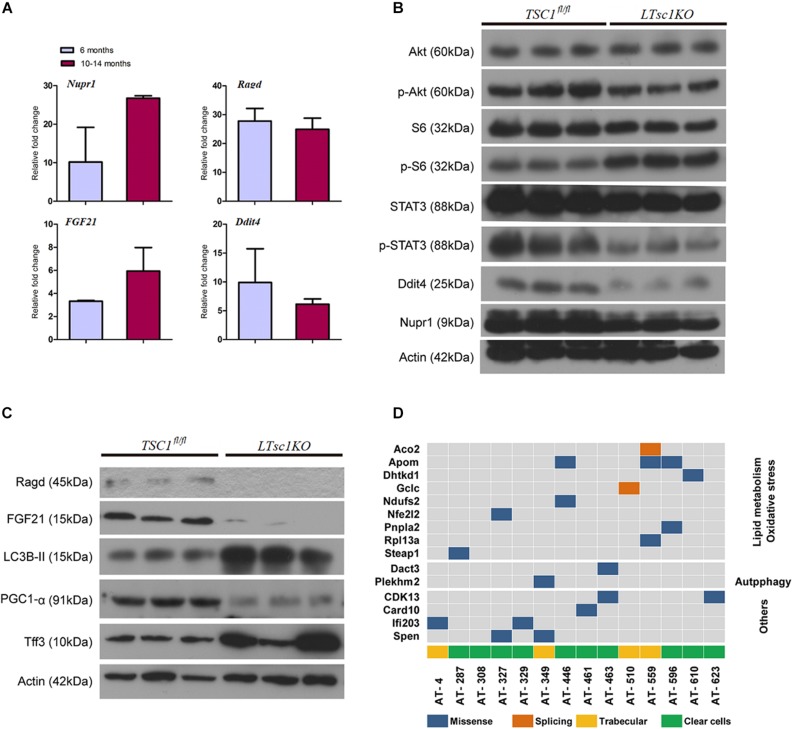
Liver carcinogenesis subsequent to defects in mTORC1 network, and autophagy in *LTsc1KO* mice. **(A)** qRT-PCR showing hepatic *Nupr1, Ragd, FGF21*, and *Ddit4* mRNA levels in *LTsc1KO* compared with *TSC1*^fl/fl^ mice aged 6 and 10–14 months (*n* = 19, ±SEM). **(B)** Immunoblot analysis of hepatic Akt, p-Akt, S6, p-S6, STAT3, p-STAT3, Ddit4, and Nupr1 in *LTsc1KO* and *TSC1*^fl/fl^ mice aged 10–14 months. **(C)** Immunoblot analysis of hepatic Ragd, FGF21, Atg5, LC3B-II, PGC1-α, and Tff3 in *LTsc1KO* and *TSC1*^fl/fl^ mice aged 10–14 months. **(D)** Representative images of mutational profile in tumor developed in *LTsc1KO* mice aged 10–14 months, by RNA-seq.

**FIGURE 6:**
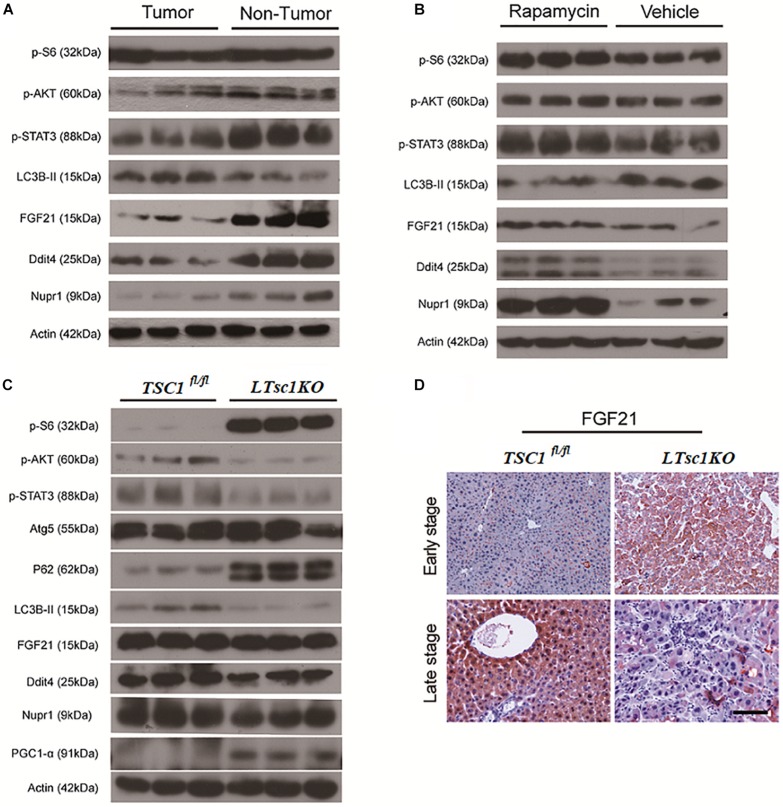
Liver Carcinogenesis Enhanced by the mTORC1–STAT Pathway Crosstalk. **(A)** Immunoblot analysis of liver lysates from tumor and non-tumor areas in *LTsc1KO* mice aged 10–14 months. **(B)** Immunoblot analysis of liver lysates from *LTsc1KO* mice, aged 10 months, treated with vehicle or rapamycin for the preceding 5 months. **(C)** Immunoblot analysis of liver lysates from *LTsc1KO* and *TSC1*^fl/fl^ mice aged 6 months. **(D)** FGF21 immunohistochemical staining of early and late stage liver sections from *LTsc1KO* and *TSC1*^fl/fl^ mice.

RNA-seq analysis identified the dysregulated genes upstream and downstream of mTORC1, including increased mRNA level of *Ragd, Ddit4, Npur1*, and *FGF21* (Figure 5A). Those gene changes were validated by qRT-PCR, and western blotting also revealed lower expression of Ragd, Ddit4, Npur1, and FGF21 proteins in *LTsc1KO* liver (Figures 5B,C), indicating that these proteins may undergo post-transcriptional modification. And no additional mutations in Ddit4, FGF21, and Nupr1 were identified (Figure 5D), indicating that protein mutations were not required for those genes after mTORC1 activation for liver carcinogenesis. While expression of Dddit4, FGF21, and Nupr1 was decreased in tumor compared to non-tumor tissue (Figure 6A). These results indicate that HCC development in *LTsc1KO* mice involves a complex network that requires Dddit4, FGF21, and Nupr1. Therefore, a cohort of mice aged 5 months was treated with rapamycin or NS three times a week for 5 months. Rapamycin-treated *LTsc1KO* livers showed reduced LC3B-II, and increased Ddit4 and Nupr1. NS-treated *LTsc1KO* mice developed HCC at a rate similar to our previous cohort. Blocking mTORC1 significantly rescued the decreased expression of Nupr1 and Ddit4 in *LTsc1KO* liver, indicating that the upstream regulatory network dictates the pathological consequences of dysregulated mTOR signaling in liver carcinogenesis (Figure 6B). Although autophagy-related gene 5 (Atg5) was unchanged, increased levels of LC3B-II (*Map1lc3b-II*) were observed in *LTsc1KO* compared to *TSC1*^fl/fl^ mice, we also found that livers from *LTsc1KO* mice at 6 months age displayed accumulation of the autophagy substrate p62, which is believed to target ubiquitinated proteins and is selectively degraded by autophagy. The abundance of p62 was increased in *LTsc1KO* livers (Figure 6C), indicating that mTORC1 activation by *TSC1* loss caused a defect in autophagy flux. We conclude that long-term activation of mTORC1 by *LTsc1KO*, upstream regulators (Dddit4) of mTORC1 was impaired; meanwhile, expression of downstream genes (Nupr1 and FGF21) of mTORC1 was decreased, resulting in metabolic disorder and mitochondrial defects.

### Dysregulation of FGF21 Was a Later Event for Liver Carcinogenesis in *LTsc1KO* Mice

There was no obvious change in expression of FGF21 in *LTsc1KO* mice treated with rapamycin, indicating that altered expression of FGF21 might be a critical event after long-term abrogation of Ddit4 and Nupr1 for HCC development (Figure 6B). Unchanged expression of FGF21 and decreased expression of Ddit4 and Nupr1 were confirmed in *LTsc1KO* mice at 6 months, indicating that FGF21 expression was an essential event during liver carcinogenesis (Figure 6C). FGF21 protein level increases in liver tissues at an early stage, but decreases in liver tissues later when HCC develops (Figure 6D). Loss of FGF21 may play an important role in HCC carcinogenetic transformation during metabolic liver injury in *LTsc1KO* mice. Metabolic remodeling associated with FGF21 expression also requires induction of liver-integrated stress-response-driven Nupr1 (Maida et al., 2016). In the present study, Nupr1 was decreased in *LTsc1KO* mice by 10–14 months, indicating its involvement in maintaining mitochondrial defects, thereby contributing to the lipotoxic effects of liver carcinogenesis. mTORC1 negatively regulates hepatic FGF21 expression via peroxisome proliferator-activated receptor-γ coactivator 1α (PGC-1α) (Estall et al., 2009). mTORC1 also controls the transcriptional activity of PGC1-α, which induces mitochondrial biogenesis in the liver (Puigserver, 2005). At 6 months of age, mice without spontaneous HCC revealed increased PGC-1α in *LTsc1KO* mice (Figure 6C). Conversely, at 10–14 months of age, PGC-1α was markedly reduced in mice with HCC (Figure 5C). Our data also confirm that altered expression of FGF21 and Nupr1 is required during liver carcinogenesis.

### Crosstalk Between mTORC1 and the STAT3 Pathway Was Linked to Non-inflammatory HCC in *LTsc1KO* Mice

Ddit4 is a constant modulator of p-Akt in response to growth factors and nutrients (Dennis et al., 2014), and its expression depends on activation of STAT3 (Pinno et al., 2016). In the present study, *LTsc1KO* mice demonstrated reduced expression of p-Akt, p-STAT3 and Ddit4 (Figure 5B). Therefore, we posit that Ddit4 might act as a mediator of crosstalk between mTOR and the STAT pathway. Correspondingly, lower expression of p-STAT3 were observed in *LTsc1KO* mice at 6 months, and *LTsc1KO* mice exhibited lower expression of p-STAT3 in tumor compared to non-tumor tissue. Rapamycin-treated *LTsc1KO* livers showed increased p-STAT3 expression compared to livers from NS-treated *LTsc1KO* mice. We therefore propose that the crosstalk between mTORC1 and the STAT3 pathway and/or other mutations was involved in non-inflammatory HCC development.

## Discussion

Given that mTORC1 regulates glucose homeostasis, lipid metabolism and cell proliferation, it is not surprising that mTOR plays a pivotal role in HCC development, and TSC1/2 mutations define a molecular subset of HCC with aggressive behavior (Ho et al., 2017). However, mechanisms of mTORC1 activation-associated HCC are not well understood. It has become necessary to understand the consequences of mTORC1 activation. To address this important issue, we aimed to provide insight into the mechanisms of HCC development in *LTsc1KO* mice, which is analogous to human HCC induced by NAFLD without necroinflammation and cirrhosis.

In the present study, the histopathology revealed two major types of tumor that were morphologically consistent with clear cell HCC and trabecular HCC in *LTsc1KO* livers. Gege expression analysis demonstrated the expression of many genes in the *LTsc1KO* liver that are also induced in human HCC and showed a specific molecular characteristics similar to non-cirrhotic DEN-mediated HCC (Degasperi and Colombo, 2016). Relationships between steatosis, steatohepatitis, cirrhosis and HCC are not necessarily linear and this pattern possibly applies to HCC arising in non-alcoholic, non-cirrhotic liver disease (Baffy et al., 2012). In NAFLD-associated HCC, there was a 23–50% prevalence of tumors developing in non-cirrhotic livers in Japan, Italy and the US (Yasui et al., 2011; Dyson et al., 2014; Mittal et al., 2015; Piscaglia et al., 2016). Therefore, in patients with NAFLD, HCC can arise in the context of non-cirrhotic liver, suggesting a specific carcinogenic pathway. Here, *LTsc1KO* mice provide us with an optimal model to investigate the underlying molecular mechanisms of steatohepatitic HCC development in the absence of inflammation.

HCC is often initiated by death of hepatocytes, resulting in liver injury followed by inflammation. The production of pro-tumorigenic cytokines including IL-6, which induces STAT3 activation in hepatocytes, eventually promotes compensatory proliferation in hepatocytes that have escaped cell death, and subsequently, tumor development. Hepatic deletion of genes in the PI3K–Akt–mTOR pathway, including Pten and Akt1 in Akt2, induces HCC (Horie et al., 2004; Watanabe et al., 2005; Kenerson et al., 2013; Wang et al., 2016). For example, hepatic Pten deletion results in hepatic injury and cell death, which activates tumor-initiating cells to induce HCC development (Galicia et al., 2010). Both hepatic Akt1 and Akt2 deletion mice develop HCC, which is associated with liver injury and inflammation via activated STAT3 and IL-6 expression (Wang et al., 2016). Menon et al. (2012) described the liver damage, inflammation, necrosis and regeneration in HCC developed in *LTsc1KO* mice. The most intriguing and unexpected observation that was uncovered in our study was spontaneous HCC in *LTsc1KO* mice without long-term hepatic injury, and inflammation. The number of infiltrative lymphocytes and macrophages (Kupffer cells), and the level of cytokine remained unchanged, in the *LTsc1KO* mice. Moreover, decreased Akt without IL-6 elevation in *LTsc1KO* mice, due to a negative regulation loop, suggested that mTORC1 activation was independent of Akt for HCC development. We revealed a previously unappreciated role for dysregulated mTORC1 signaling in promoting cancer-initiating events via inhibition of STAT3. Previous research suggested that STAT3 was activated in inflammatory HCC. It has been proved that increased IL-6 production results in activation of the oncogenic transcription factor STAT3 in liver, and TNF-α is a major adipose- derived cytokine and potent activator of pro-oncogenic pathways, including mTOR. Hepatocyte-specific ablation of the specific mTORC1 subunit Raptor resulted in elevated IL-6 production, activation of STAT3, and enhanced HCC development (Umemura et al., 2014). It is worth noting that mice with hepatic deletion of STAT5 are more susceptible to hepatocarcinogenesis, and the hepatic deletion of STAT5 induces STAT3 activity (Hosui et al., 2009). In the current study, we found that STAT5 is highly expressed (data not shown) and thus may inhibit the expression of STAT3. Perhaps, this is evidence to support our findings that mTORC1 might have a negative regulatory effect on STAT3.

Obesity, hepatosteatosis, insulin resistance, and chronic mTORC1 activation are associated with a pronounced increase in HCC risk (Calle et al., 2003). Our findings elucidate fundamental biochemical properties displayed by mTORC1 activation to achieve their tumor-promoting effect. There is an increasing appreciation of the fact that oncogenic signaling controls the metabolic reprogramming of cancer cells (Yecies and Manning, 2011). mTORC1, a master regulator of cellular metabolism, controls lipogenesis and glucose metabolism, however, the mechanisms and critical factors need to be elucidated. It is unclear whether the altered fatty acid composition observed in the *LTsc1KO* liver contributes to tumorigenesis.

The transformation of chemicals is important in carcinogenesis the Cyp450 families are key enzymes in tumor transformation, and mediate the metabolic activation of numerous pre-carcinogens, such as Cyp1a2 and Cyp2e1, which degrade xenobiotics, steroids and fatty acids. By integrated omics profiling, we incorporated enzyme transcript levels into stearic acid accumulation. We hypothesized that metabolic disorder accompanied with inactivation of xenobiotic metabolizing enzymes, and causes defective necrosis, apoptosis and autophagy, in addition to chronic mTORC1 activation in promoting anabolic growth and proliferation.

Transformed cells survive by acquiring adaptations that allow mTORC1 to continue signaling and are insensitive to the stress of energy, oxygen and nutrient deprivation, which confers a selective growth advantage. Cancer evolves via a multistep process and molecular events in addition to TSC1 deficiency during tumor development. Kenerson et al. (2013) described that phosphorylation of InsR was reduced in TSC1-deficiency tumors, which is upstream of mTORC1. A gene network containing FGF21, Ddit4, and Nupr1 could explain at least in part the transition consistent with activation of an adaptive transcriptional survival program.

**FIGURE 7:**
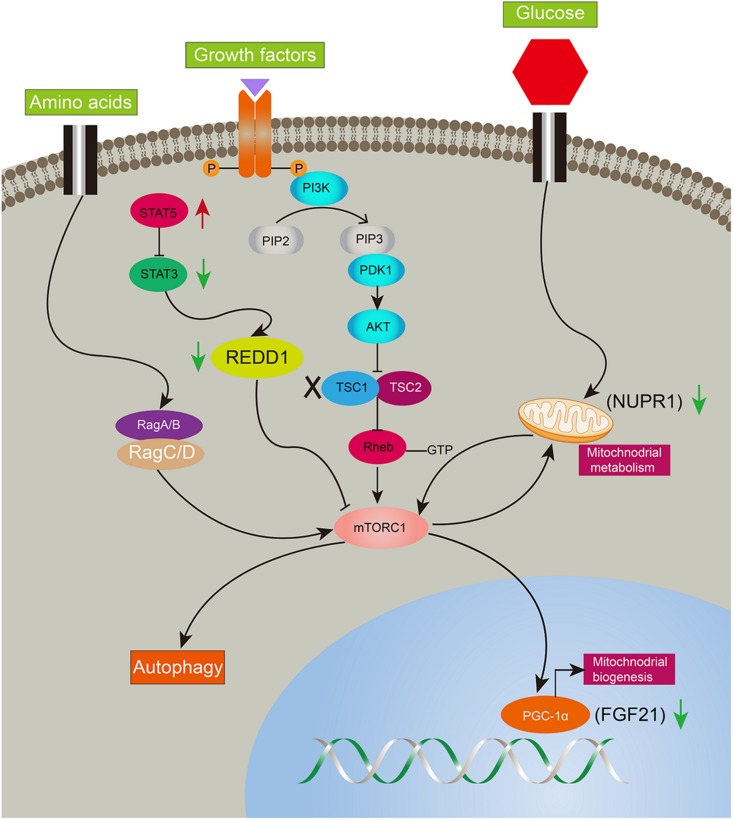
Illustration of complicated gene network of consecutive mTORC1 activation in *LTsc1KO* mice. Long-term activation of mTORC1 in *LTsc1KO* mice. Negative upstream regulators of mTORC1 were shut off, such as Dddit4, meanwhile, expression of Nupr1, and FGF21 downstream of mTORC1 were decreased for mitochondrial defects, autophagy defect and oxidative stress. Red arrow, increase; green arrow, decrease.

Ddit4 also named Redd1, regulated in development and DNA damage responses-1, is an mTORC1 inhibitor and critical transducer of the cellular response to energy depletion (Sofer et al., 2005). Abnormalities of Ddit4-mediated signaling promote tumorigenesis (Ellisen, 2005; Pineau et al., 2010). Moreover, Ddit4 expression depends on the activation of STAT3 (Pinno et al., 2016). In the present study, Ddit4 was decreased and reversed after rapamycin given, indicating mTORC1 negative feedback on Ddit4. Nupr1, a mitochondrial defect-responsive gene, regulates autophagy induced by the lipotoxic effects of excess fatty acid accumulation in cells (Jia et al., 2016). Nupr1 has also been identified as a key regulator and metabolic switch in response to mitochondrial damage during liver cancer progression (Lee et al., 2015). In the present study, Nupr1 was decreased in *LTsc1KO* mice by 10–14 months, while mTORC1 negatively regulates hepatic FGF21 expression via PGC-1α. TSC1 deficiency abrogates FGF21-mediated inhibition of mTORC1 (Gong et al., 2016). Overexpression of hepatocyte-specific FGF21 could prevent DEN-induced liver carcinogenesis in transgenic mice (Huang et al., 2006). Mice lacking FGF21 develop substantial fatty liver and markedly exacerbated accumulation of liver triglycerides, consistent with impaired fatty acid oxidation (Fisher et al., 2014). Ddit4 is also an emerging link for crosstalk between mTOR and STAT pathways in HCC development, and highlights that consecutive STAT5 activation may partially protect 4E-BP1 phosphorylation (Nogami et al., 2015). Insufficiency of Nupr1 in *LTsc1KO* mice results in non-sensitivity to a variety of stressors, including respiratory deficiency. Loss of FGF21 worsens metabolic disorder and contributes to aberrant molecular events, including lipid metabolism, in HCC development. The dysregulated expression of FGF21 is a later and critical event for HCC development. Furthermore, metabolic remodeling associated with FGF21 expression also requires induction of liver-integrated stress-response-driven Nupr1 (Maida et al., 2016). Therefore, our data favor the possibility that *LTsc1KO* mice constructed a regulator network and crosstalk between mTOR and STAT3 has been attributed to oxidative, ER stress, mitochondrial dysfunction, and autophagy defects, adding a potential mechanism to induce and maintain constitutive activation of mTORC1, and mediate a metabolic feature for growth advantage (Figure 7).

**FIGURE 8:**
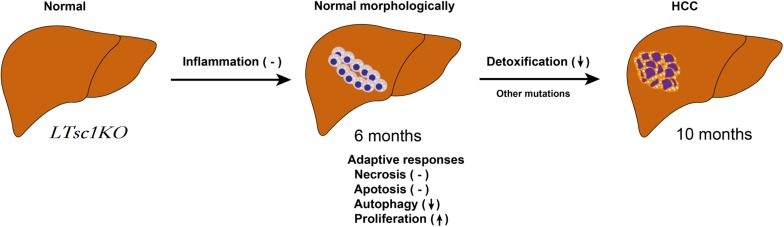
Proposed Mechanism of HCC Development in *LTsc1KO* Mice. mTORC1 activation results in loss of control over four key homeostatic responses in hepatic cells, comprising mitochondrial defects, detoxification loss and autophagy inhibition, and dysregulation of these adaptive responses (necrosis, apoptosis, autophagy, and proliferation) contribute to HCC development. This model give rise to HCC with gene signatures similar to human chemical HCC.

In summary, we unraveled a comprehensive gene network and obtained mechanistic insights into how mTORC1 activation as a primary driver results in loss of control over four key homeostatic responses in hepatic cells, comprising metabolic disorder, mitochondrial defect, detoxification loss, and autophagy inhibition. mTORC1 activation, Ddit4 and STAT3 phosphorylation inhibition, indicatingdysregulation of Ddit4-mTORC1-STAT3 lop ultimately contributes to development of non-inflammatory HCC (Figure 8). The HCC that developed in *LTsc1KO* mice in this study provides a typical animal model for future investigation into the molecular events underlying carcinogenesis arising from non-cirrhotic liver disease.

## Data Availability Statement

All datasets generated for this study are included in the article/Supplementary Material.

## Ethics Statement

All procedures involving mice was approved by the Southern Medical University Animal Care and Use Committee.

## Author Contributions

TL, GZ, XX, and YG designed the experiments. TL generated mice and conducted the most experiments, with assistance from LW. LW and SL made the pathological evaluations of the tissue sections. TL and GZ analyzed all the data, completed the experiments for publication, and wrote the paper. YG conceived the project, and secured funding.

## Conflict of Interest

The authors declare that the research was conducted in the absence of any commercial or financial relationships that could be construed as a potential conflict of interest.

## Supplementary Material

FIGURE S1 Consequences of mTORC1 Activation by *LTsc1KO*. **(A)** Representative PCR genotyping of *Alb* (left) and *TSC1* (right). Left: lane 1 and 3: *Alb-Cre* positive, lane 2 and 4: *Alb-Cre* negative, lane 5: water, lane 6: DNA maker 100 bp ladder. Right: lane 1: DNA double-chain *TSC1-loxp* site insertion, lane 2 and 4: no *TSC1-loxp* site insertion, lane 3: DNA single-chain *TSC1-loxp* site insertion, lane 5: water, lane 6: DNA maker 100 bp ladder. *Alb-Cre* recombinase is a site-specific enzyme, could catalyze recombination between two *TSC1-loxp* sites, delation the *TSC1* gene. Take together, lane 1 is *Alb-TSC1*^fl/fl^ mice, also refers to *LTsc1KO* mice, lane 2 and 4 is *TSC1 ^+/+^* mice, also refers to C57BL/6j mice, lane 3 is *Alb-TSC1*^fl/+^ mice. **(B)** (Related to Figure 1). Representative pictures of livers in 10–14 months old *LTsc1KO* and *TSC1*^fl/fl^ mice. **(C)** (Related to Figure 2). Hepatic levels of inflammatory cytokines quantified in 10–14-month-old *LTsc1KO* and *TSC1*^fl/fl^ mice, including IL-3, IL-5, IL-12(P40), IL-12(P70), macrophage inflammatory protein (MIP)1α, MIP1β, interferon-γ, and monocyte chemotactic protein 1 (*n* = 19, ±SEM).

TABLE S1 Primer sequences used for RT-PCR.

## Abbreviations

mTOR: mammalian target of rapamycin
mTORC1: Mammalian target of rapamycin complex 1
*LTsc1KO*: liver special TSC1 knockdown
HCC: hepatocellular carcinoma
NAFLD: nonalcoholic fatty liver disease
TSC1: tuberous sclerosis-1
DEN: diethylnitrosamine
AKT: protein kinase B
Ddit4: DNA-damage-inducible transcript 4
Nurp1: nuclear protein 1
FGF21: fibroblast growth factor 21
γH2AX: Phosphorylated histone-2AX
ALT: alanine transaminase
AST: aspartate transaminase
TNF: tumor necrosis factor
GPX3: glutathione peroxidase 3
CYP450: cytochrome P450
Ces: carboxylesterases
Sults: sulfotransferases
Ugts: UDP-glucuronosyltransferases
Atg5: autophagy-related gene 5
PGC-1α: peroxisome proliferator-activated receptor PPAR-γ coactivator 1α
STAT3: signal transducer and activator of transcription 3.
