# Supplementary figures and images for: Defects in mTORC1 Network and mTORC1-STAT3 Pathway Crosstalk Contributes to Non-inflammatory Hepatocellular Carcinoma
Source: Front Cell Dev Biol. 2020 Apr 7;8:225. doi: 10.3389/fcell.2020.00225 (PMC7182440; doi:10.3389/fcell.2020.00225)

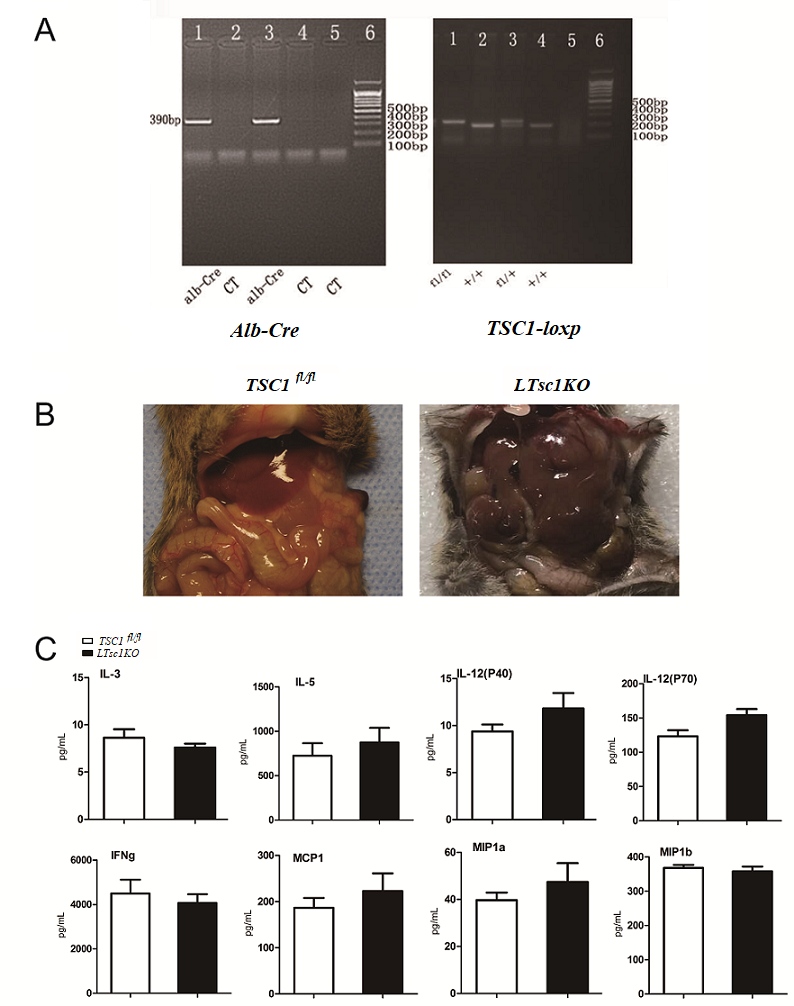

Supplement: FIGURE S1 — Consequences of mTORC1 Activation by LTsc1KO. (A) Representative PCR genotyping of Alb (left) and TSC1 (right). Left: lane 1 and 3: Alb-Cre positive, lane 2 and 4: Alb-Cre negative, lane 5: water, lane 6: DNA maker 100 bp ladder. Right: lane 1: DNA double-chain TSC1-loxp site insertion, lane 2 and 4: no TSC1-loxp site insertion, lane 3: DNA single-chain TSC1-loxp site insertion, lane 5: water, lane 6: DNA maker 100 bp ladder. Alb-Cre recombinase is a site-specific enzyme, could catalyze recombination between two TSC1-loxp sites, delation the TSC1 gene. Take together, lane 1 is Alb-TSC1fl/fl mice, also refers to LTsc1KO mice, lane 2 and 4 is TSC1 +/+ mice, also refers to C57BL/6j mice, lane 3 is Alb-TSC1fl/+ mice. (B) (Related to Figure 1). Representative pictures of livers in 10–14 months old LTsc1KO and TSC1fl/fl mice. (C) (Related to Figure 2). Hepatic levels of inflammatory cytokines quantified in 10–14-month-old LTsc1KO and TSC1fl/fl mice, including IL-3, IL-5, IL-12(P40), IL-12(P70), macrophage inflammatory protein (MIP)1α, MIP1β, interferon-γ, and monocyte chemotactic protein 1 (n = 19, ±SEM). [file Image_1.tif]
