# Supplementary material for: Defects in mTORC1 Network and mTORC1-STAT3 Pathway Crosstalk Contributes to Non-inflammatory Hepatocellular Carcinoma
Source: Front Cell Dev Biol. 2020 Apr 7;8:225. doi: 10.3389/fcell.2020.00225 (PMC7182440; doi:10.3389/fcell.2020.00225)
Supplement: TABLE S1 — Primer sequences used for RT-PCR. [file Data_Sheet_1.docx]

**Supplemental Information**

**Supplementary materials and methods**

**Tumor Quantification**

Microscopically visible tumors were measured as previously described ([Menon et al., 2012](#_ENREF_1)).

**Histopathology, Histochemistry and Immunocytochemistry**

The livers were fixed in 4% paraformaldehyde and embedded in paraffin for further histopathology, histochemistry and immunocytochemistry analyses. Paraffin sections (4–5 μm) were subjected to H&E staining. Inflammatory cell infiltration was measured by examination of H&E-stained liver sections. To detect glycogen accumulation, PAS staining was performed as previously described ([Murakata et al., 2000](#_ENREF_2)). Sirius red staining was performed to detect fibrosis as previously described ([Umemura et al., 2014](#_ENREF_3)). Immunohistochemistry staining was performed as previously described ([Umemura et al., 2014](#_ENREF_3)). The primary antibodies included anti-Ki67 (Cell Signaling Technology, Danvers, MA, USA), anti-F4/80 (Abcam, Cambridge, MA, UK), anti-cleaved caspase 3 (Cell Signaling Technology), anti-p-STAT3 (Abcam), and anti-FGF21 (Abcam).

**Measurement of Glucose**

Glucose levels were determined by Catalyst DxTM Chemistry Analyzer (Yeeran Technology Ltd., Beijing, China).

**Measurement of the liver enzymes AST and ALT**

Serum levels of ALT and AST were determined by Catalyst DxTM Chemistry Analyzer (Yeeran Technology Ltd., Beijing, China).

**Cytokines, Chemokines and Growth Factors**

Cytokines, chemokines and growth factors were analyzed at Oebiotech Enterprise (Shanghai, China), by quantitative cytokine assays using Bio-Plex mouse cytokine 23-plex panel based on xMAP technology (Bio-Rad Laboratories, Hercules, CA, USA) according to the pre-optimized protocol.

**RNA-seq and Bioinformatic Analysis**

We extracted total RNA using the RNeasy Plus Mini Kit (Qiagen, Valencia, CA, USA). cDNA library construction and illumine HiSeq4000 sequencing were conducted at Novogene Bioinformatics Institute (Beijing, China). Original RNA-seq data were deposited in the NCBI Gene Expression Omnibus (GEO GSE147354). Bioinformatics analysis was carried out by Novogene bioinformatics and DAVID GO was performed using the online accessible DAVID database (http://david.abcc.ncifcrf.gov).

**Real-time qRT-PCR**

RNA (1 μg) was used to generate cDNA with PrimeScrip RT Master Mix (Perfect Real Time) (TaKaRa Biotechnology, Japan). Individual gene expression was quantified by StepOnePlus Real-Time PCR System (Life Technologies) and ChamQ SYBR Color qPCR Master Mix (High ROX Premixed) (Vazyme Biotech, Nanjing, China) and normalized to a housekeeping control gene, *β-actin*. Primer sequences for real-time RT-PCR are available upon request and listed in the table below.

| **Table1. Primer sequences used for RT-PCR** | | |
| --- | --- | --- |
| Gene | Forward Primer (5'-3') | Reverse Primer (5'-3') |
| *Tff3* | TTGCTGGGTCCTCTGGGATAG | TACACTGCTCCGATGTGACAG |
| *Spink1* | TTTGGCCCTGCTGAGTTTAG | GCCCACCTTTTCGAATGAGG |
| *Ly6d* | GGTTCCGAGGTCACACAATG | CAGAGAGCCATAACAGTGAGC |
| *Gpc3* | CAGCCCGGACTCAAATGGG | CAGCCGTGCTGTTAGTTGGTA |
| *Afp* | TGTCATTGCAGATTTCTCAGGC | GACTCGTTTTGTCTTCTCTTCCC |
| *Cd44* | TCCTCTAGCAGTTCTTTGACAGA | CAAGGTAAGAGGTTCAGAGGTGA |
| *Rnase1* | CTGCAACCAAATGATGAAACGC | CCTTCAGGTGGCAGTCAGTG |
| *H19* | CTTGTCGTAGAAGCCGTCTGTTC | GTAGCACCATTTCTTTCATCTTGAGG |
| *Bex2* | TCCAAAGTGGAACAAGGCGT | GCACGTAGTAGTCTCCAGCTTC |
| *Nupr1* | CCCTTCCCAGCAACCTCTAAA | TCTTGGTCCGACCTTTCCGA |
| *Ragd* | CTGTTTGACGTGGTCAGTAAGAT | GTTGAGTCCTTGTCATACGGG |
| *FGF21* | GTGTCAAAGCCTCTAGGTTTCTT | GGTACACATTGTAACCGTCCTC |
| *Ddit4* | CAAGGCAAGAGCTGCCATAG | CCGGTACTTAGCGTCAGGG |
| *β-actin* | CCTGAGGCTCTTTTCCAGCC | TAGAGGTCTTTACGGATGTCAACGT |

**Immunoblotting**

Liver tissue was homogenized and lysed in RIPA buffer containing phosphatase inhibitors and protease inhibitor cocktail. Solubilized proteins were collected by centrifugation and quantified using a BCA protein assay kit (Thermo Scientific). Equal amounts of protein (50 μg) from each sample were resolved by electrophoresis in an 8%, 10% or a 12% gel and transferred to polyvinylidene difluoride membranes (Bio-Rad). The primary antibodies included anti-Akt (Cell Signaling Technology), anti-P-Akt (Cell Signaling Technology), anti-S6K (Cell Signaling Technology), anti-PS6K (Cell Signaling Technology), anti-actin (Sigma–Aldrich), anti-cleaved caspase 3 (Cell Signaling Technology), anti-p53 (Cell Signaling Technology), anti-STAT3 (Abcam), anti-p-STAT3 (Cell Signaling Technology), anti-GPX3 (Abcam), anti-LC3B-II (Cell Signaling Technology), anti-Ddit4 (Abcam), anti-Nupr1 (Bioss), anti-FGF21 (Abcam), anti-Tff3 (Thermo Fisher Scientific), anti-PGC1-α (Abcam), anti-p62 (Abcam).

**ELISA**

Serum GPX3 levels were measured using ELISA (BioTSZ, San Francisco, CA, USA). OD values were collected by Synergy HT (BioTek, Shoreline, WA, USA).

**Metabolomics**

Sample preparation and metabolomic profiling were performed in collaboration with Shanghai Biotree Biotech Co. Ltd. The tissue metabolic profiling analysis was conducted by gas chromatography time-of-flight mass spectrometry.

**Reference**

Menon, S., Yecies, J.L., Zhang, H.H., Howell, J.J., Nicholatos, J., Harputlugil, E., et al. (2012). Chronic activation of mTOR complex 1 is sufficient to cause hepatocellular carcinoma in mice. *Sci Signal* 5(217)**,** ra24. doi: 10.1126/scisignal.2002739.

Murakata, L.A., Ishak, K.G., and Nzeako, U.C. (2000). Clear cell carcinoma of the liver: a comparative immunohistochemical study with renal clear cell carcinoma. *Mod Pathol* 13(8)**,** 874-881. doi: 10.1038/modpathol.3880156.

Umemura, A., Park, E.J., Taniguchi, K., Lee, J.H., Shalapour, S., Valasek, M.A., et al. (2014). Liver damage, inflammation, and enhanced tumorigenesis after persistent mTORC1 inhibition. *Cell Metab* 20(1)**,** 133-144. doi: 10.1016/j.cmet.2014.05.001.
